# Trends in health care of patients with vasculitides, including giant cell arteritis, Takayasu arteritis, ANCA-associated vasculitis and Behçet’s disease: cross-sectional data of the German National Database 2007–2021

**DOI:** 10.1007/s00296-023-05508-x

**Published:** 2024-01-05

**Authors:** Jörg Henes, Jutta G. Richter, Katja Thiele, Uta Kiltz, Johanna Callhoff, Katinka Albrecht

**Affiliations:** 1 Centre for Interdisciplinary Clinical Immunology, Rheumatology and Autoimmune Diseases and Internal Medicine II, University Hospital Tübingen, Tübingen, Germany; 2 Department of Rheumatology, Faculty of Medicine, University Hospital Düsseldorf, Heinrich-Heine-University Düsseldorf, Düsseldorf, Germany; 3 Faculty of Medicine, Hiller Research Centre Rheumatology, University Hospital Düsseldorf, Heinrich-Heine-University Düsseldorf, Düsseldorf, Germany; 4 Programme Area Epidemiology and Health Services Research, German Rheumatism Research Centre Berlin, Berlin, Germany; 5 Ruhr Universität Bochum, Rheumazentrum Ruhrgebiet, Herne, Germany; 6 Institute of Social Medicine, Epidemiology and Health Economics, Charité-Universitätsmedizin Berlin, Berlin, Germany

**Keywords:** Giant cell arteritis, Takayasu arteritis, Granulomatosis with polyangiitis, Microscopic polyangiitis, Behçet’s disease, Health care

## Abstract

The aim of this study is to present the current care situation of patients with giant cell arteritis (GCA), Takayasu arteritis (TAK), ANCA-associated vasculitis (AAV) and Behçet’s disease (BD). Trends over the last 15 years will reflect improvements and remaining deficits in the management of vasculitides. Consecutive cross-sectional data from patients with vasculitides from the German National Database (NDB) of the Collaborative Arthritis Centres between 2007 and 2021 were included. Medication, physician- and patient-reported outcomes on disease activity and disease burden, inpatient stays and occupational participation are compared for different vasculitis entities and over time. Employment rates were compared to German population rates. Between 502 and 854 vasculitis patients were annually documented. GCA and AAV were the most common vasculitides. Median disease duration ranged from 2 to 16 years. Over the years, glucocorticoids decreased in proportion and dose, most markedly in GCA and TAK, while biologic therapies increased up to 27%. Physicians rated disease activity as low for the vast majority of patients, while patients-reported moderate outcomes in many dimensions. PROs remained largely unchanged. The proportion of employed patients (< 65 years) increased from 47 to 57%. In recent years, biologics are increasingly used in patients with vasculitides, while glucocorticoids decreased significantly. PRO’s have not improved. Work participation increased but remains lower than that in the German population.

**Supplementary Information:**

The online version contains supplementary material available at 10.1007/s00296-023-05508-x.

## Introduction

Vasculitides are systemic inflammatory rheumatic diseases (iRMD) whose common feature is the autoimmune inflammation of the blood vessels. Vessels of different sizes can be affected individually or in combination. According to the 2012 Chapel-Hill nomenclature [1], vasculitides are classified into large vessel (Giant cell arteritis, GCA, and Takayasu arteritis, TAK), medium-sized-vessel, small-vessel (ANCA-associated vasculitides, AAV) and variable-vessel vasculitides (including Behçet’s disease, BD). Updated classification criteria have recently been developed for GCA [2], TAK [3] and ANCA subtypes [4–6] to create more specific criteria for classification and to recognize phenotypic overlap between entities [7]. Vasculitides affect all age groups, although the various forms differ significantly in the age of onset. While some vasculitides occur in childhood (IgA vasculitis, Kawasaki syndrome), other forms manifest predominantly in younger adults (BD, TAK), in middle age (AAV) or above the age of 50 (GCA).

Vasculitides are rare diseases with worldwide prevalence rates of 20–250 per 100,000 persons for GCA [8] and 18.7–21.0 per 100,000 for AAV [9] as the most common forms. In a regional German survey, 40–49/100,000 persons aged above 50 years had GCA and 13–17/100,000 had AAV [10]. Recent claims data provided a higher prevalence with 26/100,000 for AAV [11]. Both studies contributed to a current estimate of approximately 35,000 people affected from those two vasculitis forms in Germany [12]. The prevalence of BD is reported with 0.6–0.8/100,000 in people of German origin and 40/100,000 in people of Turkish origin in Germany [13]. Prevalence data on TAK is available for other European countries with 1.3/100,000 in Sweden and 1.3–3.3/100,000 in Turkey [14].

Within the spectrum of internal medicine and rheumatology, patients with vasculitides represent the smallest disease entities, but due to the severity and complexity of vasculitides, they require a high level of interdisciplinary care. Until recent years, glucocorticoids (GCs) were indispensable for the management of vasculitides as conventional therapeutics often failed to achieve sufficient immunosuppression. Within the last 10 years, the first biologics have been approved for various forms of vasculitis. The increasing therapeutic options offer a better achievement of remission and saving potential of GCs.

Due to the rare occurrence and the heterogeneous vasculitis entities, very little data are available on the long-term health care situation of patients with vasculitides. The German National Database (NDB) of the Collaborative Arthritis Centres annually collects physician- and patient-reported data since 1993. The NDB is not disease specific to vasculitides, but its generic approach captures many aspects of vasculitides that are relevant to the health-related quality of care [15]. The aim of this study is to present the current care situation of patients with different vasculitides. This includes medication, physician- and patient-reported outcomes on disease activity and disease burden, inpatient stays and work participation. Trends over the last 15 years shall reflect improvements and remaining deficits in the management of vasculitides.

## Methods

The National Database of the German Collaborative Arthritis Centres (NDB) is an ongoing prospective long-term monitoring study. It was established in 1993 as an instrument for monitoring the health care of iRMDs. The NDB contains clinical data and patient-reported outcomes for unselected outpatients with iRMDs. Nationwide, rheumatology centres collect data on patients with IRDs in routine care. The NDB has been specifically conceptualized to provide data on healthcare for all patients with iRMDs. [15]. Every year, around 12,000 patients with iRMDs are documented, of which about 800 have vasculitis. Physician- and patient-reported data are annually collected and include clinical data on disease activity, medication and comorbidities as well as patient-reported outcomes such as disease impact and work participation.

### Patients

Inclusion criteria: all patients with a confirmed vasculitis diagnosis who participated in the NDB between 2007 and 2021 were included. It is facultative to participate every year. Cross-sectional data of all patients documented in the respective years were taken into account. Diagnoses were documented by the rheumatologists and classified according to ICD-10GM coding: GCA (M31.5,6), TAK (M31.4), Granulomatosis with polyangiitis (GPA, M31.3), Eosinophilic granulomatosis with polyangiitis (EGPA, M30.1), Microscopic polyangiitis (MPA, M31.7), BD (M35.2), Panarteritis nodosa (PAN, M30.0,8), Anti-GBM disease (Goodpasture’s syndrome, M31.0), IgA vasculitis (D69.0), Cryoglobulinemic vasculitis (D89.1), Hypocomplementaemic urticarial vasculitis (M31.8) and unspecified vasculitides. Patients with suspected diagnosis were not considered.

Sociodemographic information includes age, sex, disease duration and symptom duration at the time of diagnosis. Comorbidities are documented by the rheumatologists at the first visit and are updated at each visit. They are classified into the following groups according to their ICD-10GM coding: arterial hypertension, coronary or other heart disease, diabetes mellitus, thyroid disease, respiratory/lung disease, neoplasia, osteoporosis, depression or mental illness, chronic pain syndrome/fibromyalgia, liver disease, peripheral arterial disease and stroke or cerebral circulation disorder.

### Main outcome variables

Physicians annually documented the current immunomodulatory therapy including GC, azathioprine, ciclosporin A, cyclophosphamide, methotrexate, mycophenolate, rituximab, TNF inhibitors, tocilizumab and apremilast. They assessed the patients’ current disease activity (recorded with a numerical rating scale (NRS) from 0 to 10, for all NRS: 0: no, 10: highest disease activity). Patients self-reported their global health status, disease activity, pain, fatigue, sleeping disorders, function, coping, emotional and physical well-being (all on NRS 0–10), adapted from the Rheumatoid Arthritis Impact of Disease (RAID) questionnaire [16]. Well-being was assessed with the WHO-5 (0–100), which was categorized into 3 groups: good (> 50), moderate (29–50) or poor well-being (< 29) [17].

All participants were asked about hospital stays due to the rheumatic disease in the last 12 months and the number of days spent in hospital.

Information on employment were documented in patients < 65 years and include full- or part-time work, unemployment, retraining/education, early retirement and sick leave due to the rheumatic disease in the last 12 months with the number of days on sick leave.

### Procedures

Ethics approval was obtained from the ethics committee of the Charité–Universitätsmedizin Berlin (EA1/196/06). This research was conducted in agreement with the Declaration of Helsinki.

### Statistical analysis

Current data from 2021 are presented for the different vasculitis entities GCA, TAK, GPA, EGPA, MPA and BD and include sociodemographics, comorbidities, medication, physician-and patient-reported outcomes, hospitalization and employment. These are reported for the AAV sub-entities GPA, EGPA and MPA, if case numbers were *n* > 30, and for the total group of vasculitides including all entities.

Trends over time are reported for patient characteristics, medication, physician-and patient-reported outcomes, hospitalization and employment for the most common vasculitis entities GCA, TAK, AAV and BD. Trends in annual employment and early retirement due to vasculitis are presented for the total group of vasculitides for patients < 65 years of age (former official retirement age in Germany), stratified by sex. Employment rates are displayed in comparison to German population data by sex in the age group 15 to under 65 years [18].

## Results

### Patient characteristics

In 2021, 854 of 11,826 patients in the NDB (7.2%) had a vasculitis. The most common form was GCA (36%), followed by AAV (28%), BD (19%) and TAK (4.3%), refer to Table 1. Among AAV, GPA was most common (68%) compared to EGPA (17%) and MPA (15%). Other vasculitis diagnoses were panarteritis nodosa (*n* = 18), hypocomplementaemic vasculitis (*n* = 9), anti-GBM disease (*n* = 3), cryoglobulinemic vasculitis (*n* = 3), IgA vasculitis (*n* = 2) and unspecified vasculitides (*n* = 69).

**Table 1:** Patient characteristics and immunomodulating therapies in 2021

	GCA	TAK	AAV				BD	All vasculitides (other forms included)
			All	GPA	EGPA	MPA		
*N*	306	37	241	164	40	37	166	854
Age, mean in years ± SD	73.3 ± 8.5	48.3 ± 16.0	61.0 ± 13.6	61.8 ± 13.5	58.5 ± 12.2	59.8 ± 15.2	43.8 ± 11.8	61.1 ± 16.4
Disease duration, mean in years	5.7 ± 5.5	11.8 ± 7.8	11.0 ± 8.1	11.9 ± 8.5	10.6 ± 7.1	7.4 ± 6.0	14.8 ± 9.2	9.6 ± 8.2
Female (%)	77	92	53	49	58	68	41	61
Medication, *N* (%)								
Glucocorticoids	152 (50)	15 (41)	156 (65)	103 (63)	30 (75)	23 (62)	78 (47)	450 (53)
Of those with GCs, dose > 5 mg/d	48 (32)	1 (7)	31 (13)	18 (18)	7 (23)	6 (27)	13 (17)	107 (24)
Methotrexate	94 (31)	3 (8.1)	67 (28)	46 (28)	12 (30)	9 (24)	16 (10)	209 (25)
Azathioprine	9 (3.0)	7 (19)	66 (27)	42 (26)	13 (33)	11 (30)	38 (23)	140 (17)
Mycophenolate	0 (0)	1 (2.7)	12 (5)	10 (6.1)	1 (2.5)	1 (2.7)	0 (0)	16 (1.9)
Ciclosporin A	0 (0)	0 (0)	0 (0)	0 (0)	0 (0)	0 (0)	5 (3.0)	7 (0.8)
Cyclophosphamide	0 (0)	0 (0)	5 (2.1)	4 (2.4)	1 (2.5)	0 (0)	0 (0)	11 (1.3)
Biologic therapy	83 (27)	14 (38)	67 (28)	50 (31)	6 (15)	11 (30)	36 (22)	227 (27)
Anakinra	1 (0.3)	0 (0)	2 (0.8)	2 (1.2)	0 (0)	0 (0)	0 (0)	5 (0.6)
Tocilizumab	79 (26)	11 (30)	0 (0)	0 (0)	0 (0)	0 (0)	0 (0)	95 (11)
TNFi	3 (1.0)	2 (5.4)	1 (0.4)	1 (0.6)	0 (0)	0 (0)	35 (21)	45 (5)
Rituximab	0 (0)	0 (0)	42 (17)	45 (27)	2 (5.0)	11 (30)	1 (0.6)	72 (8.5)
Apremilast							11 (6.7)	

*GCA* Giant cell arteritis, *GPA* granulomatosis with polyangiitis, *EGPA* eosinophilic granulomatosis with polyangiitis, *MPA* microscopic polyangiitis, *SD* standard deviation

The mean age was 44 years (BD), 48 years (TAK), 49 years (GPA), 58 years (EGPA), 68 years (MPA) and 73 years (GCA). The proportion of women was highest in TAK (92%) and GCA (77%), followed by MPA (68%), EGPA (58%), GPA (49%) and BD (41%).

Comorbidities are displayed in supplementary Fig. 1. The main findings were osteoporosis (24%) in GCA, thyroid disease (29%), peripheral artery disease (14%) and stroke/cerebral circulatory disorder in TAK (5.7%), concomitant lung disease in EGPA (65%), depression (21%) and chronic pain/fibromyalgia (12%) in BD.

### Medication

GCs were used in 41% (TAK) to 75% (EGPA). Overall, 24% had a GC dose above 5 mg/d, most frequently in GCA (32%). Methotrexate and azathioprine were the most commonly used conventional synthetic DMARDs. Of all vasculitides patients, 27% had a bDMARD therapy. These were in particular tocilizumab in GCA (30%) and TAK (30%), rituximab in GPA (27%) and MPA (30%), and TNF inhibitors in BD (21%). Of all BD patients, 6.7% received apremilast (Table 1).

### Physician- and patient-reported outcomes

For all vasculitides, physicians rated the disease activity as low in 92% of patients, while only 58% of patients rated their disease activity as low but moderate in 30% and high in 12%. Only 41% (GCA) to 57% (EGPA) rated their global health as good. According to the WHO-5, good well-being was reported by 56% of all patients, ranging from 44% (TAK) to 59% (EGPA). Mean values for pain, fatigue, coping, function, sleeping disorders and well-being ranged between 2.5 ± 2.8 (mean ± standard deviation) and 4.6 ± 3.3 with worse values for fatigue and sleeping disorders in MPA and better values for pain and coping in GPA and EGPA (Table 2).

**Table 2:** Physician- and patient-reported outcomes in 2021

	GCA	TAK	GPA	EGPA	MPA	BD	Total*
Physician-reported disease activity (NRS 0–10), mean	1.3 ± 1.6	1.5 ± 1.4	1.5 ± 1.4	1.3 ± 1.1	1.5 ± 1.4	1.2 ± 1.3	1.3 ± 1.4
No/mild (0–3), *N* (%)	271 (90.3)	33 (91.7)	152 (93.3)	39 (97.5)	34 (91.9)	156 (94.0)	780 (92.4)
Moderate (4–6)	27 (9.0)	3 (8.3)	10 (6.1)	1 (2.5)	3 (8.1)	10 (6.0)	60 (7.1)
Severe (7–10)	2 (0.7)	0 (0)	1 (0.6)	0 (0)	0 (0)	0 (0)	4 (0.5)
Patient-reported disease activity (NRS 0–10), mean	3.5 ± 2.5	3.1 ± 2.7	3.2 ± 2.3	3.3 ± 2.4	3.5 ± 2.7	3.4 ± 2.6	3.3 ± 2.5
No/mild (0–3), *N* (%)	144 (55.0)	16 (55.2)	85 (63.0)	21 (63.6)	18 (56.3)	59 (55.1)	397 (57.6)
Moderate (4–6)	84 (32.1)	10 (34.5)	36 (26.7)	7 (21.2)	8 (25.0)	32 (29.9)	208 (30.2)
Severe (7–10)	34 (13.0)	3 (10.3)	14 (10.4)	5 (15.2)	6 (18.8)	16 (15.0)	84 (12.2)
Patient global health (NRS 0–10), mean	4.3 ± 2.1	4.0 ± 2.1	3.9 ± 2.2	3.5 ± 2.3	4.1 ± 2.5	4.0 ± 2.5	4.1 ± 2.2
Good (0–3), *N* (%)	110 (40.7)	15 (48.4)	63 (45.0)	21 (56.8)	18 (50.0)	54 (50.0)	318 (44.5)
Moderate (4–6)	111(41.1)	12 (38.7)	62 (44.3)	12 (32.4)	11 (30.6)	35 (32.4)	285 (39.9)
Poor (7–10)	49 (18.1)	4 (12.9)	15 (10.7)	4 (10.8)	7 (19.4)	19 (17.6)	111 (15.5)
WHO-5 (0–100, 0: worst)							
Good Well-being (> 50), *N* (%)	142 (56.6)	11 (44.0)	74 (56.9)	19 (59.4)	17 (53.1)	47 (52.8)	357 (55.5)
Moderate well-being (29–50)	48 (19.1)	7 (28.0)	27 (20.8)	0 (0)	6 (18.8)	15 (16.9)	122 (19.0)
Poor well-being (≤ 28)	61 (24.3)	7 (28.0)	29 (22.3)	13 (40.6)	9 (28.1)	27 (30.3)	164 (25.5)
Fatigue, mean (SD)	3.8 ± 2.8	4.1 ± 3.1	3.7 ± 2.7	3.8 ± 2.9	4.3 ± 3.1	4.2 ± 3.3	3.9 ± 2.9
Pain, mean (SD)	3.3 ± 2.6	3.0 ± 2.6	2.6 ± 2.4	2.5 ± 2.8	3.3 ± 2.9	3.2 ± 2.8	3.0 ± 2.6
Sleeping disorders, mean (SD)	3.8 ± 3.1	3.7 ± 3.1	3.4 ± 3.1	4.1 ± 3.5	4.6 ± 3.3	4.1 ± 3.4	3.3 ± 3.2
Function, mean (SD)	3.4 ± 2.7	3.2 ± 2.9	3.2 ± 2.7	2.6 ± 2.6	3.2 ± 3.1	3.2 ± 3.1	3.3 ± 2.8
Physical well-being, mean (SD)	3.9 ± 2.4	3.6 ± 2.5	3.4 ± 2.3	3.3 ± 2.5	4.0 ± 2.6	3.8 ± 2.9	3.7 ± 2.5
Emotional well-being, mean (SD)	3.5 ± 2.6	3.4 ± 2.8	3.0 ± 2.5	3.2 ± 2.5	3.7 ± 2.7	3.8 ± 3.1	3.4 ± 2.7
Coping, mean (SD)	3.0 ± 2.6	2.8 ± 2.7	2.5 ± 2.4	2.6 ± 2.7	3.3 ± 3.1	3.1 ± 3.0	2.9 ± 2.6

*GCA* Giant cell arteritis, *GPA* granulomatosis with polyangiitis, *EGPA* eosinophil granulomatosis with polyangiitis, *MPA* microscopic polyangiitis, *NRS* numeric rating scale, *SD* standard deviation

*Including all other rare vasculitides

### Hospitalization and work participation

Between 11% (TAK) and 20% (GCA) of patients were hospitalized in the past year with a median duration of 14 (Interquartile range (IQR) 5; 15) days, being longest in AAV patients.

From all patients < 65 years, 57% were employed, 63% of men and 53% of women. Thereof 38% were in full time and 20% in part-time employed. Sick leave due to the vasculitis in the past year was present in 30% with a median number of 15 (IQR 10; 180) days. Early retirement due to vasculitis was present in 15% (Table 3).

**Table 3:** Hospitalization and work participation in 2021

	GCA	TAK	AAV	BD	All vasculitides (other forms included)
Hospitalization, *N*	264	28	204	107	697
Hospitalized in the past 12 months, *N* (%)	53 (20.1)	3 (10.7)	37 (18.1)	12 (11.2)	136 (19.5)
Number of days in hospital, median (1st Quartile; 3rd Quartile)	10 (5; 14)	*	14 (6, 21)	7 (3, 14)	14.0 (5, 15)
Employment					
Number of patients < 65 years	46	23	112	98	339
Employed	22 (47.8)	12 (52.2)	65 (58.0)	60 (61.2)	194 (57.2)
Full time	14 (30.4)	5 (22)	44 (39.3)	42 (43)	127 (37.5)
Part time	8 (17.4)	7 (30)	21 (18.8)	18 (18)	67 (19.8)
Male *N* employed/*N* male (%)	4/7 (*)	0/0	35/55 (63.6)	33/53 (62.3)	90/143 (62.9)
Female *N* employed/*N* female (%)	18/39 (46.2)	12/23 (52.2)	30/57 (52.6)	27/45 (60.0)	104/196 (53.1)
Incapacity to work due to vasculitis in the past 12 months, *N* (%)	10 (45.5)	5 (45.5)	14 (22.6)	13 (24.1)	55 (29.9)
Number of days in sick leave, median (1st Quartile; 3rd Quartile)	16 (14; 350)	10 (*)	25 (10; 180)	10 (7; 112)	15 (10; 180)
Early retirement due to vasculitis, *N* (%)	7 (15)	6 (26)	19 (17)	12 (12)	52 (15)

*GCA* Giant cell arteritis, *GPA* granulomatosis with polyangiitis, *EGPA* eosinophil granulomatosis with polyangiitis, *MPA* microscopic polyangiitis, *NRS* numeric rating scale, *SD* standard deviation

*Case number is too low to show this value

#### Trends over time

### Patient characteristics

From 2007 to 2021, the number of vasculitis patients per year varied between 502 and 854. Within all IRDs, the proportion of vasculitis increased from 3.9% in 2007 to 7.2% in 2021. The proportion of GCA increased over the course, while AAVs tended to decrease. With a stable age of onset, the mean age at the time of the survey increased by 2 to 4 years for GCA, AAV and BD. The age of onset for TAK decreased to the late 30 s. The mean symptom duration until diagnosis was stable (from 2014) for GCA and AAV. In BD patients, the above-average duration of symptoms has decreased (mean 3.9 to 2.8 years). Patient characteristics for each year are reported in supplementary Table 1.

### Trends in medication

**Fig. 1:**
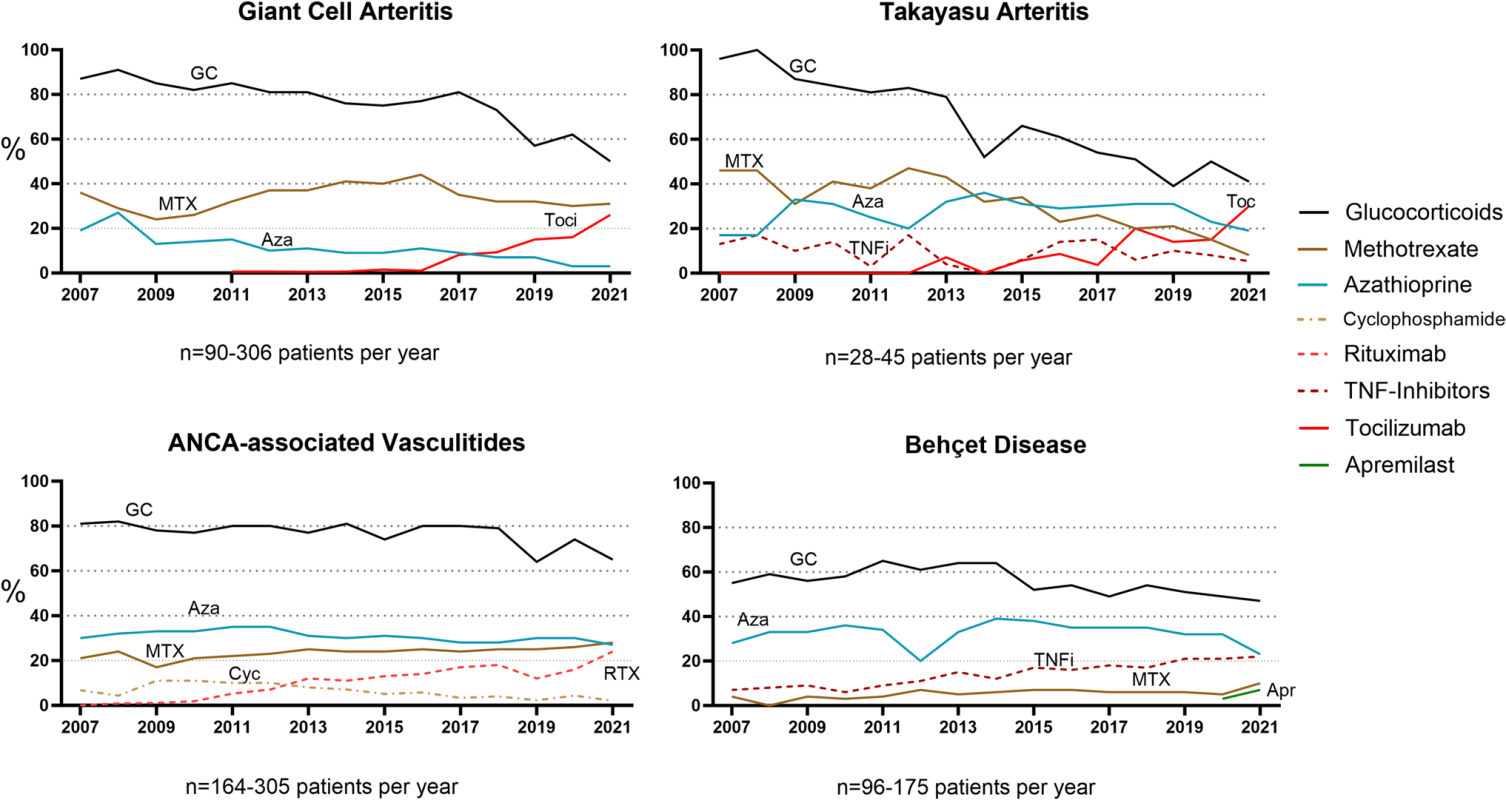
Trends in immunomodulating therapies in patients with vasculitides from the German National Database between 2007 and 2021

Overall GC use decreased in all vasculitides, most markedly in GCA (87% to 50%) and TAK (96% to 41%), but also in AAV (81% to 65%) and BD (55% to 47%). The proportion of patients with GC > 5 mg/day also decreased in GCA (46% to 16%), TAK (70% to 7%), AAV (42% to 13%) and BD (40% to 17%). Biologic therapies increased from 4.5% to 24% regarding all vasculitides. Rituximab was first used for AAV in 2008 and increased from 0.9% to 24%. Tocilizumab was first given for GCA in 2011 and increased from 0.6% to 26% in GCA and 30% in TAK. TNF inhibitors were increasingly used in BD (6.9% to 21%). Methotrexate and azathioprine were less frequently used in subsequent years (Fig. 1).

### Trends in patient-reported outcomes and in physician-reported disease activity

**Fig. 2:**
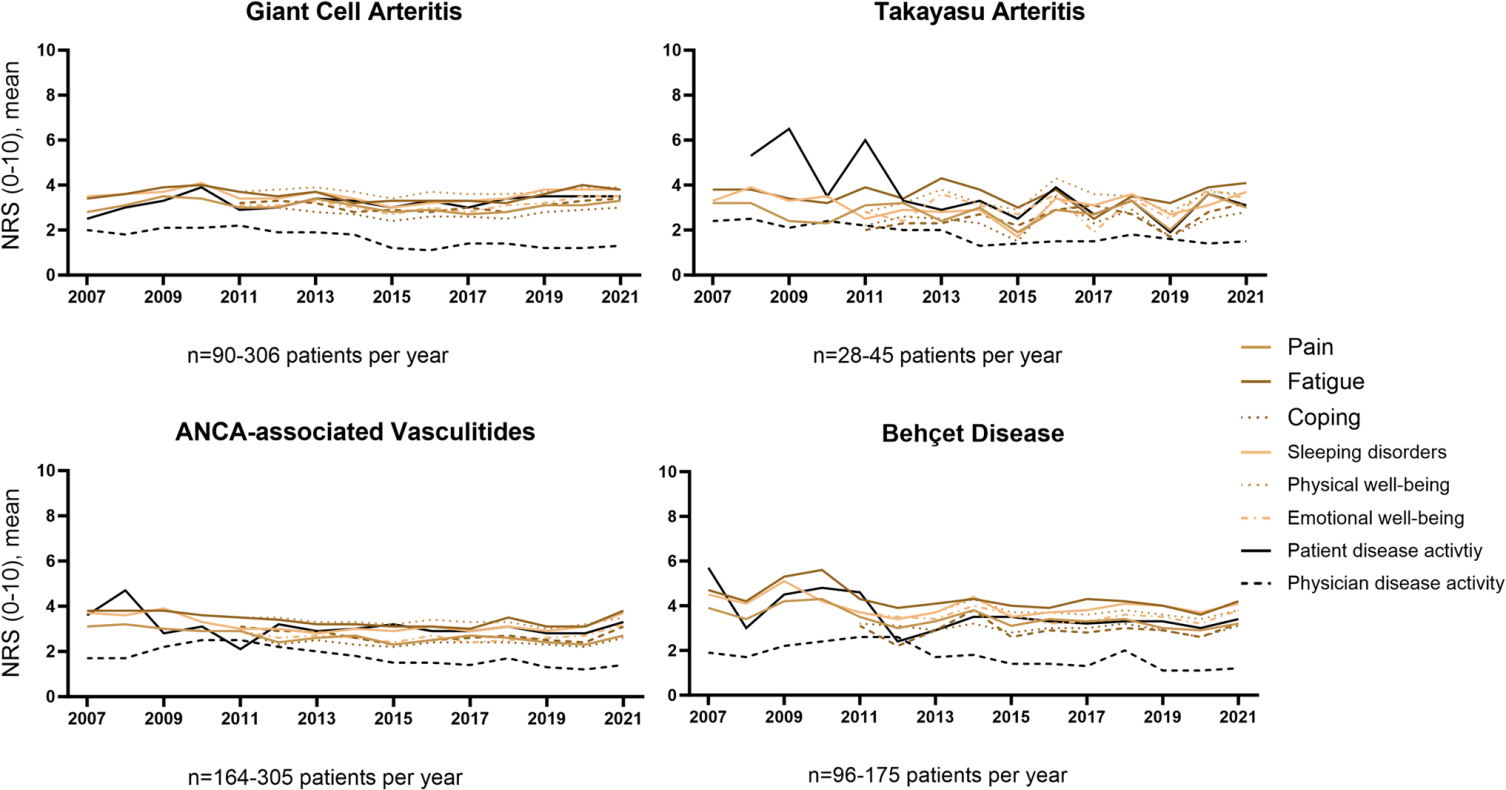
Trends in patient-reported outcomes and physician-reported disease activity between 2007 and 2021

Across all vasculitides, the mean physician-reported disease activity was low in all years (1–2 out of 10 NRS points), while the mean patient-reported disease activity was 1–2 points higher in each year. The other PROs such as pain, fatigue, coping, function and well-being showed very comparable trends to the patient-reported disease activity and did not improve over the years (Fig. 2).

### Trends in hospitalization and work participation

**Fig. 3:**
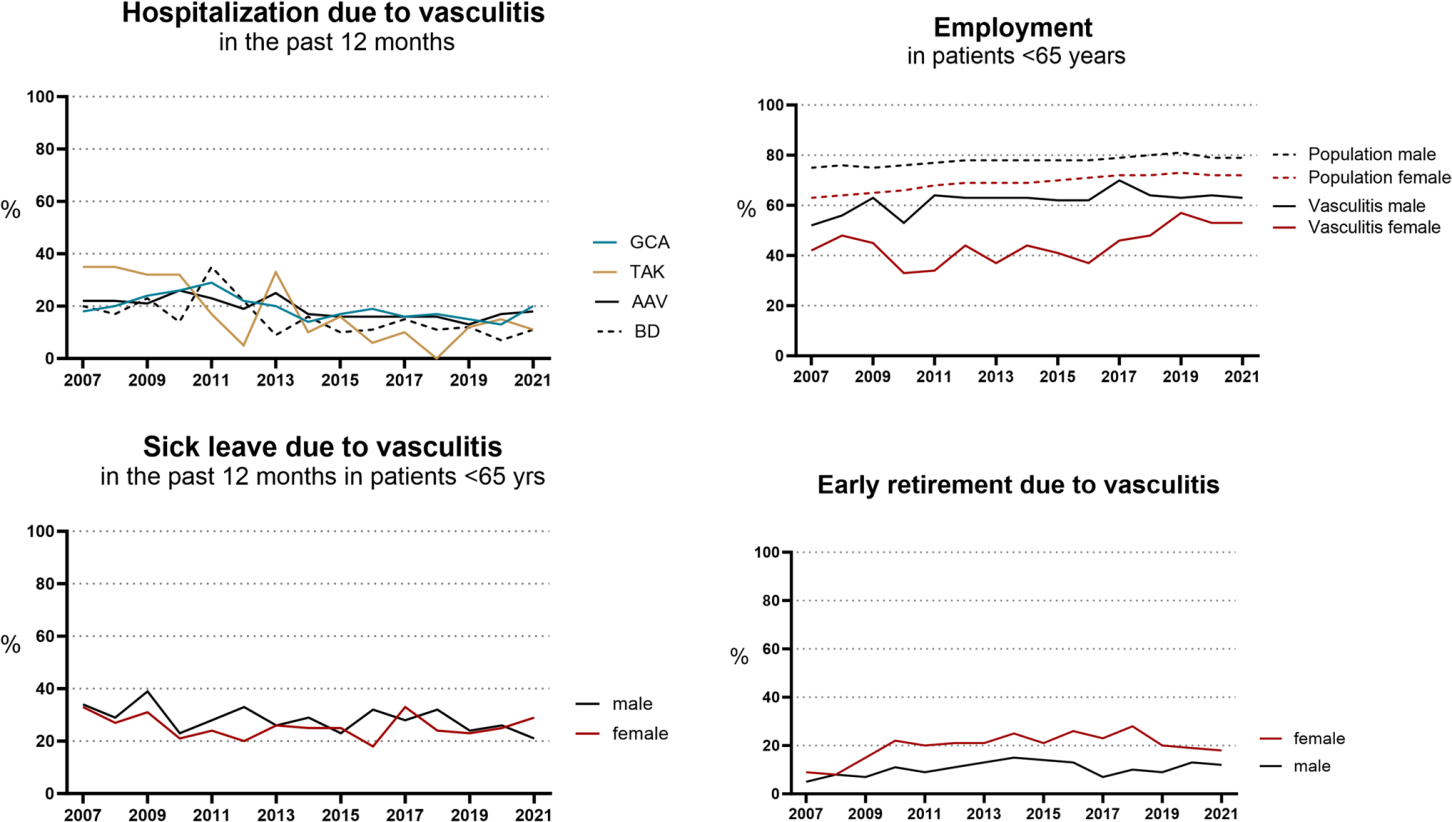
Trends in hospitalization, sick leave, employment and early retirement between 2007 and 2021. GCA Giant cell arteritis, *TAK* Takayasu arteritis, *AAV* ANCA-associated vasculitides, *BD* Behçet’s disease. Population data derived from [18] https://www.destatis.de accessed 2023 04 26

Hospitalization and sick leave have declined overall with fluctuations in the individual years. Employment increased from 47% in 2007 to 57% in 2021. Men had consistently higher employment rates compared to women. Compared to population data, the employment rate over the entire period was lower in women and men with vasculitis, in 2021 by 19% in women and 16% in men. The proportion of early retirement due to vasculitis was 7% in 2007, rose to 20% in 2014 and declined again to 15% in 2021. Women more frequently had early retirement compared to men (Fig. 3).

## Discussion

The annual data from the NDB of the last 15 years show developments in therapy and health care of patients with established vasculitides. The recent approval of various biologics for individual vasculitis entities has significantly changed the treatment spectrum. In the same period in which biologics were increasingly used, the overall intake of GCs could be reduced which is beneficial for the patients for various reasons (e.g. risk for infections, other side effects).

Our reported distribution of vasculitis diagnoses corresponds to expectations with GCA and AAV as the most common forms. The slight increase in the proportion of GCA patients may be due to the increasing age of patients among others as a result of demographic change. Slightly declining AAV proportions are probably due to chance, there is no rationale for this. The proportion of AAV subgroups corresponds to recent data from the European ANCA-associated vasculitis registries [19]. The high proportion of BD is caused by a special consultation for BD in one of the participating centres. The gender distribution shows, comparable to other European cohorts, the highest proportion of women in TAK [20], more women than men with GCA [21], more men than women with BD [22] and a relatively balanced ratio in AAV [19, 23–25]. Considerable differences in the age of onset, ranging from 28 (BD) to 67 years (GCA), are indicated. The average disease duration of the NDB vasculitis patients is more than two years; thus, the results should be considered as care outcomes under maintenance therapy. It is noticeable that BD patients had a mean symptom duration of more than 3 years and waited longer than average for a diagnosis. A delay in diagnosis of several years is also reported in other studies [26]. However, the trend indicates a shorter symptom duration in the more recent years.

Trends in medication show an increasing use of biologics in all vasculitides. The large-vessel vasculitides GCA and TAK have been increasingly treated with tocilizumab since the 2017 approval for RZA. At the same time, an impressive decrease in glucocorticoids can be observed. Methotrexate continues to be used as an alternative, as it is recommended in the EULAR recommendations [27]. TNF inhibitors, azathioprine and mycophenolate, are used in TAK. AAVs are frequently treated with rituximab, which has been approved for GPA and MPA since 2013. In the European vasculitis registries, rituximab maintenance therapy was used in 16%, with large variation in the single registries [19]. In AAV, GCs are also declining, especially in dose, but continue to be included in long-term therapy for more than half of the patients. There is little change in conventional immunosuppressants with azathioprine, methotrexate and mycophenolate for AAVs, and cyclophosphamide is being used less frequently. Mepolizumab (approval at the end of 2021 for EGPA) and avacopan (approval at the beginning of 2022 for GPA and MPA), both of which are already included in the new EULAR recommendations [28], are today considered as treatment options alongside rituximab; for these drugs, data are not available in the NDB yet. TNF inhibitors are increasingly used in patients with BD since the approval of adalimumab in 2016 for the treatment of uveitis. Other BD therapies include mainly azathioprine but also ciclosporin A and methotrexate. Glucocorticoids have been declining here more slowly. Apremilast was approved in 2020 for the treatment of patients with oral aphthae [29] and was prescribed in 6.7% of BD patients in 2021. There are only little data on medication of vasculitis from routine care in Germany. Monocentric cohort studies from the 1990s and 2000s already showed improved life expectancy in patients with GPA [24], EGPA [30] and MPA [31], but all were still treated with conventional immunosuppressants. The Joint Vasculitis Registry (GeVas) was initiated in 2019 to prospectively record the course of vasculitis after initial diagnosis or after therapy changes in case of relapses [32]. The first current data were presented in 2022 for the treatment of GCA [33]. Of the 131 patients, mostly with initial diagnosis, all received GC therapy, 49% tocilizumab, 19% MTX and 19% cyclophosphamide. In contrast to the NDB, the GeVas data reflect the remission-inducing therapies as an inception cohort, which explains the differences in therapies.

The predominantly low disease activity assessed by physicians indicates that most patients are in the stage of a well-controlled disease under immunomodulatory therapy. The patient’s assessment is higher, a discrepancy that is also known from other inflammatory rheumatic diagnoses [34, 35], and has not improved over the years. It can be assumed that the physician assessment is based on other parameters (laboratory parameters, organ involvement) than the patient assessment. A study by Rode et al. confirms to this assumption showing a high association between physician-reported disease activity and increased CRP values as well as shorter disease duration in contrast to a high association between patient-reported disease activity and patient-reported limitations regarding pain, function and general well-being in patients with AAVs [23]. Hurtado-Arias et al. found correlations between patient global assessment and social and emotional impact, physical function and concerns about the future in AAVs [36]. The annual comparisons of this study show very similar trends in the individual PRO dimensions at the same level as the patient-reported disease activity. In the course of the Outcome Measure Development in Large-vessel Vasculitis (OMERACT), pain, fatigue and emotional impact have been identified as important outcomes in large-vessel vasculitis [37]. The long-term data on these PROS from the NDB contribute to the evidence and show that we need to pay more attention to PROs in the management of vasculitides.

Work participation has increased in the last 15 years in women and men, especially in women in the 2010s. However, both rates are still below the employment rate in the population and are also significantly lower than for patients with rheumatoid arthritis or spondyloarthritis within the NDB [38]. This indicates that the severity of the underlying disease of vasculitis and the resulting damage still leads to restrictions in occupational participation, even with satisfactory disease control. Hospital stays are more frequent and with a longer duration compared to patients with RA (6–14% per year, median 9–13 days) [38].

The comorbidity spectrum of vasculitides differs significantly. Apart from age-related differences, associated strokes remain a relevant concern in young TAK patients. In AAV patients, secondary diseases are common, especially in the organs frequently affected by vasculitis [19]. In the case of BD, the high proportion of depression should be mentioned, which was already shown in a monocentric study by Saur et al. with a clear difference to healthy controls [39].

### Limitations and strengths

The NDB does not represent the nationwide care situation, even though the participating facilities currently originate from nine different federal states. A strength, however, is the simultaneous documentation of patients treated in private practices, outpatient hospitals and university care. Vasculitis-specific organ manifestations (including damage), disease activity biomarkers, and data on mortality and infectious diseases are not recorded in the NDB. Instead, there is broad information on treatments and PROs as well as on the health situation of patients and their participation. The greatest benefit of the NDB is the long-term documentation spanning decades, so that changes in routine rheumatological care can be mapped very well.

## Conclusions

Since the introduction of highly effective bDMARD therapies, the disease situation for patients with vasculitides has improved. The proportion of patients who are in a state of low disease activity is high and the use of glucocorticoids has been reduced, so that both disease-related and drug-induced secondary damage may be avoided or reduced. Occupational participation has risen continuously even if it does not yet reach population rates. On the other hand, many patient-reported dimensions regarding their state of health have not improved. Future studies on patient-reported outcomes in vasculitis should focus on whether chronicity of pain, sleeping disorders, fatigue and impaired well-being can be prevented by earlier specific therapies and/or whether these impairments need to be addressed by a multi-dimensional management including non-pharmacological treatment options.

### Supplementary Information

Below is the link to the electronic supplementary material.

Supplementary file1 (DOCX 235 KB)

## Data Availability

All data relevant to the study are included in the article or uploaded as online supplemental information.

## References

[1] Jennette JC, Falk RJ, Bacon PA, Basu N, Cid MC, Ferrario F, Flores-Suarez LF, Gross WL, Guillevin L, Hagen EC, Hoffman GS, Jayne DR, Kallenberg CG, Lamprecht P, Langford CA, Luqmani RA, Mahr AD, Matteson EL, Merkel PA, Ozen S, Pusey CD, Rasmussen N, Rees AJ, Scott DG, Specks U, Stone JH, Takahashi K, Watts RA (2013). 2012 revised International Chapel Hill Consensus Conference nomenclature of vasculitides. Arthritis Rheum.

[2] Ponte C, Grayson PC, Robson JC, Suppiah R, Gribbons KB, Judge A, Craven A, Khalid S, Hutchings A, Watts RA, Merkel PA, Luqmani RA, Group DS (2022). 2022 American College of Rheumatology/EULAR classification criteria for giant cell arteritis. Ann Rheum Dis.

[3] Grayson PC, Ponte C, Suppiah R, Robson JC, Gribbons KB, Judge A, Craven A, Khalid S, Hutchings A, Danda D, Luqmani RA, Watts RA, Merkel PA, Group DS (2022). 2022 American College of Rheumatology/EULAR classification criteria for Takayasu arteritis. Ann Rheum Dis.

[4] Grayson PC, Ponte C, Suppiah R, Robson JC, Craven A, Judge A, Khalid S, Hutchings A, Luqmani RA, Watts RA, Merkel PA, Group DS (2022). 2022 American College of Rheumatology/European Alliance of Associations for Rheumatology classification criteria for eosinophilic granulomatosis with polyangiitis. Arthritis Rheumatol.

[5] Robson JC, Grayson PC, Ponte C, Suppiah R, Craven A, Judge A, Khalid S, Hutchings A, Watts RA, Merkel PA, Luqmani RA, Investigators D (2022). 2022 American College of Rheumatology/European Alliance of Associations for Rheumatology classification criteria for granulomatosis with polyangiitis. Ann Rheum Dis.

[6] Suppiah R, Robson JC, Grayson PC, Ponte C, Craven A, Khalid S, Judge A, Hutchings A, Merkel PA, Luqmani RA, Watts RA, Dcvas I (2022). 2022 American College of Rheumatology/European Alliance of Associations for Rheumatology classification criteria for microscopic polyangiitis. Ann Rheum Dis.

[7] Emmi G, Vaglio A (2023). The new look of classification criteria for systemic vasculitis. Nat Rev Rheumatol.

[8] Sharma A, Mohammad AJ, Turesson C (2020). Incidence and prevalence of giant cell arteritis and polymyalgia rheumatica: a systematic literature review. Semin Arthritis Rheum.

[9] Redondo-Rodriguez R, Mena-Vazquez N, Cabezas-Lucena AM, Manrique-Arija S, Mucientes A, Fernandez-Nebro A (2022). Systematic review and metaanalysis of worldwide incidence and prevalence of antineutrophil cytoplasmic antibody (ANCA) associated vasculitis. J Clin Med.

[10] Herlyn K, Buckert F, Gross WL, Reinhold-Keller E (2014). Doubled prevalence rates of ANCA-associated vasculitides and giant cell arteritis between 1994 and 2006 in northern Germany. Rheumatology (Oxford).

[11] Hellmich B, Lamprecht P, Spearpoint P, Gotte D, Deichmann A, Buchholz I, Schonermark MP, Rutherford P (2021). New insights into the epidemiology of ANCA-associated vasculitides in Germany: results from a claims data study. Rheumatology (Oxford).

[12] Albrecht K, Binder S, Minden K, Poddubnyy D, Regierer AC, Strangfeld A, Callhoff J (2023). Systematic review to estimate the prevalence of inflammatory rheumatic diseases in Germany. German version. Z Rheumatol.

[13] Kotter I, Krusche M (2023). Inflammatory rheumatic diseases in migrants. Inn Med (Heidelb).

[14] Seyahi E (2017). Takayasu arteritis: an update. Curr Opin Rheumatol.

[15] Albrecht K, Callhoff J, Zink A (2019). Long-term trends in rheumatology care : achievements and deficits in 25 years of the German national rheumatology database. Z Rheumatol.

[16] Thiele K, Albrecht K, Zink A, Aringer M, Karberg K, Spathling-Mestekemper S, von Hinuber U, Callhoff J (2022). Is the rheumatoid arthritis impact of disease (RAID) score a meaningful instrument for other inflammatory rheumatic diseases? A cross-sectional analysis of data from the German National Database. RMD Open.

[17] Redeker I, Hoffmann F, Callhoff J, Haibel H, Sieper J, Zink A, Poddubnyy D (2018). Determinants of psychological well-being in axial spondyloarthritis: an analysis based on linked claims and patient-reported survey data. Ann Rheum Dis.

[18] Statistisches Bundesamt d (2023) Erwerbstätigenquoten 1991 bis 2022. httphttps://www.destatis.de/DE/Themen/Arbeit/Arbeitsmarkt/Erwerbstaetigkeit/Tabellen/erwerbstaetigenquoten-gebietsstand-geschlecht-altergruppe-mikrozensus.html. Accessed 26 Apr 2023.

[19] Gisslander K, Rutherford M, Aslett L, Basu N, Dradin F, Hederman L, Hruskova Z, Kardaoui H, Lamprecht P, Licholai S, Musial J, O'Sullivan D, Puechal X, Scott J, Segelmark M, Straka R, Terrier B, Tesar V, Tesi M, Vaglio A, Wandrei D, White A, Wojcik K, Yaman B, Little MA, Mohammad AJ, consortium F (2023). Data quality and patient characteristics in European ANCA-associated vasculitis registries: data retrieval by federated querying. Ann Rheum Dis.

[20] Goel R, Chandan JS, Thayakaran R, Adderley NJ, Nirantharakumar K, Harper L (2021). Cardiovascular and renal morbidity in Takayasu arteritis: a population-based retrospective cohort study from the United Kingdom. Arthritis Rheumatol.

[21] Monti S, Milanesi A, Klersy C, Tomelleri A, Dagna L, Campochiaro C, Farina N, Muratore F, Galli E, Marvisi C, Bond M, Berti A, Bortolotti R, Padoan R, Schiavon F, Felicetti M, Nannini C, Cantini F, Giollo A, Rossini M, Conticini E, Frediani B, Conti F, Priori R, Sebastiani M, Cassone G, Quartuccio L, Treppo E, Bettio S, Hoxha A, Lovisotto M, Emmi G, Mattioli I, Leccese P, Caporali R, Argolini LM, Foti R, Visalli E, Colaci M, Salvarani C, Montecucco C, Italian Society of Rheumatology Vasculitis Study G (2023). Age at diagnosis influences the clinical phenotype, treatment strategies and outcomes in patients with giant cell arteritis: results from the observational GCAGE study on a large cohort of 1004 patients. Ann Rheum Dis.

[22] Jo YG, Ortiz-Fernandez L, Coit P, Yilmaz V, Yentur SP, Alibaz-Oner F, Aksu K, Erken E, Duzgun N, Keser G, Cefle A, Yazici A, Ergen A, Alpsoy E, Salvarani C, Kisacik B, Kotter I, Henes J, Cinar M, Schaefer A, Nohutcu RM, Takeuchi F, Harihara S, Kaburaki T, Messedi M, Song YW, Kasifoglu T, Martin J, Gonzalez Escribano MF, Saruhan-Direskeneli G, Direskeneli H, Sawalha AH (2022). Sex-specific analysis in Behcet’s disease reveals higher genetic risk in male patients. J Autoimmun.

[23] Rohde M, Kernder A, Acar H, Dusing C, Fischer-Betz R, Haase I, Mucke J, Sander O, Richter J, Filla T, Schneider M, Chehab G (2023). Determinants of patient and physician global assessments of disease activity in anti-neutrophil cytoplasmic antibody-associated vasculitis. Front Med (Lausanne).

[24] Holle JU, Gross WL, Latza U, Nolle B, Ambrosch P, Heller M, Fertmann R, Reinhold-Keller E (2011). Improved outcome in 445 patients with Wegener's granulomatosis in a German vasculitis center over four decades. Arthritis Rheum.

[25] Berti A, Cornec D, Crowson CS, Specks U, Matteson EL (2017). The epidemiology of antineutrophil cytoplasmic autoantibody-associated vasculitis in Olmsted County, Minnesota: a twenty-year US population-based study. Arthritis Rheumatol.

[26] Sadeghi A, Rostami M, Amraei G, Davatchi F, Shahram F, Karimi Moghaddam A, Karimi Moghaddam Z, Zeraatchi A (2023). Clinical manifestations of Behcet’s disease: a retrospective cross-sectional study. Mediterr J Rheumatol.

[27] Hellmich B, Agueda A, Monti S, Buttgereit F, de Boysson H, Brouwer E, Cassie R, Cid MC, Dasgupta B, Dejaco C, Hatemi G, Hollinger N, Mahr A, Mollan SP, Mukhtyar C, Ponte C, Salvarani C, Sivakumar R, Tian X, Tomasson G, Turesson C, Schmidt W, Villiger PM, Watts R, Young C, Luqmani RA (2020). 2018 Update of the EULAR recommendations for the management of large vessel vasculitis. Ann Rheum Dis.

[28] Hellmich B, Sanchez-Alamo B, Schirmer JH, Berti A, Blockmans D, Cid MC, Holle JU, Hollinger N, Karadag O, Kronbichler A, Little MA, Luqmani RA, Mahr A, Merkel PA, Mohammad AJ, Monti S, Mukhtyar CB, Musial J, Price-Kuehne F, Segelmark M, Teng YKO, Terrier B, Tomasson G, Vaglio A, Vassilopoulos D, Verhoeven P, Jayne D (2023). EULAR recommendations for the management of ANCA-associated vasculitis: 2022 update. Ann Rheum Dis.

[29] Hatemi G, Mahr A, Takeno M, Kim D, Melikoglu M, Cheng S, McCue S, Paris M, Chen M, Yazici Y (2022). Impact of apremilast on quality of life in Behcet’s syndrome: analysis of the phase 3 RELIEF study. RMD Open.

[30] Moosig F, Bremer JP, Hellmich B, Holle JU, Holl-Ulrich K, Laudien M, Matthis C, Metzler C, Nolle B, Richardt G, Gross WL (2013). A vasculitis centre based management strategy leads to improved outcome in eosinophilic granulomatosis and polyangiitis (Churg-Strauss, EGPA): monocentric experiences in 150 patients. Ann Rheum Dis.

[31] Schirmer JH, Wright MN, Vonthein R, Herrmann K, Nolle B, Both M, Henes FO, Arlt A, Gross WL, Schinke S, Reinhold-Keller E, Moosig F, Holle JU (2016). Clinical presentation and long-term outcome of 144 patients with microscopic polyangiitis in a monocentric German cohort. Rheumatology (Oxford).

[32] Iking-Konert C, Wallmeier P, Arnold S, Adler S, de Groot K, Hellmich B, Hoyer BF, Holl-Ulrich K, Ihorst G, Kaufmann M, Kotter I, Muller-Ladner U, Magnus T, Rech J, Schubach F, Schulze-Koops H, Venhoff N, Wiech T, Villiger P, Lamprecht P (2021). The Joint Vasculitis Registry in German-speaking countries (GeVas) - a prospective, multicenter registry for the follow-up of long-term outcomes in vasculitis. BMC Rheumatol.

[33] Wallmeier Pass F et al. (2022) Das Gemeinsame Vaskulitis-Register im deutschsprachigen Raum (GeVas) – eine Subgruppenanalyse der 131 RZA-Patienten. Paper presented at the DGRh Berlin

[34] Barton JL, Imboden J, Graf J, Glidden D, Yelin EH, Schillinger D (2010). Patient-physician discordance in assessments of global disease severity in rheumatoid arthritis. Arthritis Care Res (Hoboken).

[35] Castrejon I, Yazici Y, Samuels J, Luta G, Pincus T (2014). Discordance of global estimates by patients and their physicians in usual care of many rheumatic diseases: association with 5 scores on a Multidimensional Health Assessment Questionnaire (MDHAQ) that are not found on the Health Assessment Questionnaire (HAQ). Arthritis Care Res (Hoboken).

[36] Hurtado-Arias JJ, Ramirez-Mulhern I, Gonzalez-Martinez C, Merayo-Chalico J, Barrera-Vargas A, Hinojosa-Azaola A (2023). Patient-reported outcomes in ANCA-associated vasculitis: a cross-sectional study to explore the interactions between patients' and physicians’ perspectives. Rheumatol Int.

[37] Aydin SZ, Robson JC, Sreih AG, Hill C, Alibaz-Oner F, Mackie S, Beard S, Gul A, Hatemi G, Kermani TA, Mahr A, Meara A, Milman N, Shea B, Tomasson G, Tugwell P, Direskeneli H, Merkel PA (2019). Update on outcome measure development in large-vessel vasculitis: report from OMERACT 2018. J Rheumatol.

[38] Thiele K, Albrecht K, Kopplin N, Callhoff J (2022) Deutsches Rheuma Forschungszentrum (DRFZ) Programmbereich Epidemiologie und Versorgungsforschung. Standardpräsentation 2020, Daten aus der Kerndokumentation. https://refubium.fu-berlin.de/handle/fub188/36566, Zugriff am 21 June 2023.

[39] Saur SJ, Schlogl A, Schmalen T, Krittian S, Pecher AC, Henes M, Xenitidis T, Henes J (2022). Sexual dysfunction and depression in Behcet’s disease in comparison to healthy controls. Rheumatol Int.

