# Supplementary material for: Trends in health care of patients with vasculitides, including giant cell arteritis, Takayasu arteritis, ANCA-associated vasculitis and Behçet’s disease: cross-sectional data of the German National Database 2007–2021
Source: Rheumatol Int. 2024 Jan 5;44(3):497–507. doi: 10.1007/s00296-023-05508-x (PMC10866751; doi:10.1007/s00296-023-05508-x)
Supplement: Supplementary file 1 — Supplementary file1 (DOCX 235 KB) [file 296_2023_5508_MOESM1_ESM.docx]

| Supplementary Table 1 Trends in patient characteristics 2007 - 2021 | | | | | | | | | | | | | | | |
| --- | --- | --- | --- | --- | --- | --- | --- | --- | --- | --- | --- | --- | --- | --- | --- |
|  | 2007 | 2008 | 2009 | 2010 | 2011 | 2012 | 2013 | 2014 | 2015 | 2016 | 2017 | 2018 | 2019 | 2020 | 2021 |
| Patients with any vasculitis diagnosis, N | 502 | 534 | 646 | 755 | 807 | 650 | 772 | 792 | 829 | 731 | 713 | 718 | 677 | 638 | 854 |
| Vasculitides among all iRMDs in the NDB (%) | 3.9 | 4.3 | 4.9 | 5.0 | 5.1 | 4.4 | 5.1 | 5.1 | 5.0 | 5.9 | 5.9 | 6.3 | 6.5 | 6.2 | 7.2 |
| **Giant Cell Arteritis,** N  (% of all vasculitides) | 90 (24) | 92 (22) | 137 (26) | 187 (30) | 209 (31) | 194 (35) | 226 (33) | 220 (32) | 229 (32) | 204 (31) | 209 (33) | 227 (35) | 208 (34) | 194 (33) | 306 (41) |
| Female (%) | 73 | 79 | 71 | 77 | 72 | 79 | 78 | 78 | 76 | 77 | 75 | 78 | 78 | 79 | 77 |
| Age, mean years | 72 | 72 | 72 | 72 | 71 | 72 | 72 | 72 | 73 | 73 | 74 | 74 | 74 | 74 | 73 |
| Age at disease onset, mean years | 68 | 68 | 68 | 68 | 67 | 67 | 68 | 67 | 68 | 68 | 68 | 68 | 68 | 68 | 67 |
| Symptom duration until diagnosis, meanyrs* | - | - | - | - | - | - | - | 0.7 | 0.7 | 0.8 | 0.7 | 0.6 | 0.8 | 0.8 | 0.8 |
| **Takayasu Arteritis,** N  (% of all vasculitides) | 28  (7) | 34  (8) | 40  (8) | 45  (7) | 37  (5) | 37  (7) | 31  (5) | 33  (5) | 37  (5) | 36  (6) | 28  (4) | 35  (5) | 29  (5) | 26  (5) | 37  (5) |
| Female (%) | 89 | 85 | 85 | 89 | 78 | 73 | 68 | 70 | 76 | 83 | 82 | 89 | 83 | 89 | 92 |
| Age, mean years | 54 | 50 | 51 | 53 | 52 | 52 | 50 | 53 | 52 | 51 | 53 | 54 | 49 | 51 | 48 |
| Age at disease onset, mean years | 45 | 44 | 44 | 45 | 42 | 42 | 43 | 43 | 42 | 41 | 41 | 41 | 38 | 39 | 37 |
| Symptom duration until diagnosis, meanyrs* | - | - | - | - | - | - | - | 1.4 | 1.6 | 1.3 | 1.0 | 1.2 | 0.9 | 1.1 | 1.7 |
| **ANCA Vasculitides**, N  (% of all vasculitides) | 164 (43) | 163 (40) | 214 (41) | 235 (37) | 259 (38) | 241 (44) | 267 (39) | 279 (41) | 305 (42) | 259 (40) | 232 (36) | 231 (36) | 223 (37) | 227 (39) | 241 (32) |
| Female (%) | 57 | 54 | 54 | 57 | 56 | 53 | 55 | 57 | 60 | 52 | 55 | 56 | 56 | 56 | 53 |
| Age, mean years | 57 | 57 | 58 | 57 | 58 | 60 | 60 | 60 | 59 | 60 | 59 | 60 | 61 | 61 | 61 |
| Age at disease onset, mean years | 49 | 49 | 50 | 50 | 50 | 50 | 51 | 50 | 49 | 49 | 48 | 49 | 50 | 50 | 50 |
| Symptom duration until diagnosis, meanyrs* | - | - | - | - | - | - | - | 1.4 | 1.4 | 1.4 | 1.4 | 1.4 | 1.1 | 1.3 | 1.4 |
| **Behçet Disease**, N  (% of all vasculitides) | 96 (25) | 122 (30) | 128 (25) | 162 (26) | 175 (26) | 78 (14) | 154 (23) | 153 (22) | 156 (22) | 152 (23) | 169 (27) | 152 (24) | 150 (25) | 134 (23) | 166 (22) |
| Female (%) | 38 | 36 | 34 | 33 | 33 | 41 | 34 | 32 | 34 | 36 | 33 | 35 | 33 | 36 | 41 |
| Age, mean years | 40 | 40 | 40 | 40 | 40 | 40 | 41 | 42 | 43 | 44 | 44 | 44 | 44 | 45 | 44 |
| Age at disease onset, mean years | 30 | 29 | 29 | 29 | 29 | 29 | 28 | 28 | 28 | 30 | 29 | 28 | 28 | 28 | 28 |
| Symptom duration until diagnosis, meanyrs* | - | - | - | - | - | - | - | 3.9 | 3.7 | 3.3 | 3.3 | 3.4 | 3.0 | 3.2 | 2.8 |
| iRMDs inflammatory rheumatic diseases, NDB German National Database of the Collaborative Arthritis Centres. *mean in years, collected since 2014 | | | | | | | | | | | | | | | |

Supplementary Figure 1 Comparison of comorbidities in patients with vasculitides in 2021


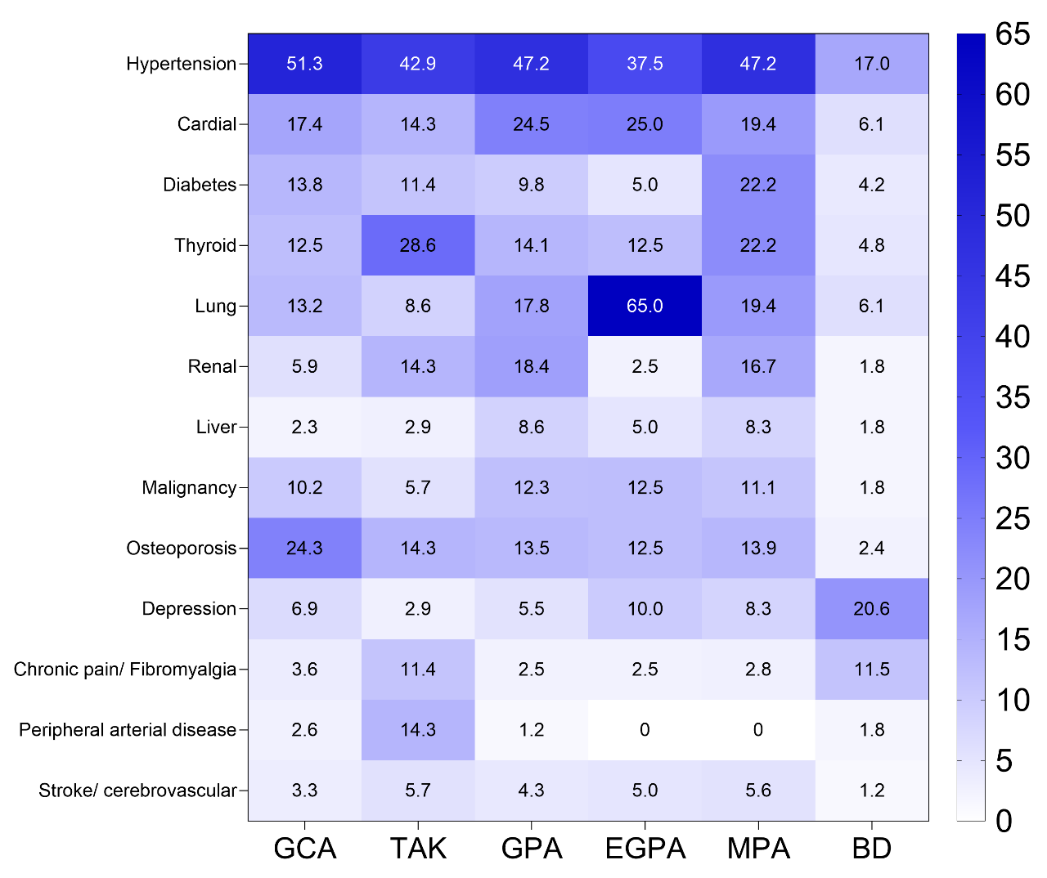


GCA Giant cell arteritis, TAK Takayasu arteritis, GPA Granulomatosis with Polyangiitis, EGPA eosinophil granulomatosis with polyangiitis, MPA microscopic polyangiitis, BD Behçet Disease.
